# Comparison between blood pressure during obstructive respiratory events in REM and NREM sleep using pulse transit time

**DOI:** 10.1038/s41598-020-60281-2

**Published:** 2020-02-24

**Authors:** Aljohara S. Almeneessier, Mana Alshahrani, Salih Aleissi, Omeima S. Hammad, Awad H. Olaish, Ahmed S. BaHammam

**Affiliations:** 1 University Sleep Disorders Center, College of Medicine, King Saud University, Riyadh, Saudi Arabia; 2 Family and Community Medicine, College of Medicine, King Saud University, Riyadh, Saudi Arabia; 3 Strategic Technologies Program of the National Plan for Sciences and Technology and Innovation in the Kingdom of Saudi Arabia, Riyadh, Saudi Arabia

**Keywords:** Hypertension, Respiratory signs and symptoms

## Abstract

Rapid eye movement-predominant obstructive sleep apnea has been shown to be independently associated with hypertension. This study aimed to non-invasively measure blood pressure during the rapid eye movement (REM) and non-rapid eye movement (NREM) obstructive events and the post-obstructive event period. Thirty-two consecutive continuous positive airway pressure-naïve obstructive sleep apnea patients (men, 50%) aged 50.2 ± 12 years underwent overnight polysomnography. Blood pressure was assessed indirectly using a validated method based on the pulse transit time and pulse wave velocity during the NREM and REM obstructive events (both apneas and hypopneas) and the post-obstructive event period. Among the recruited patients, 10 (31.3%) had hypertension. Mean apnea-hypopnea index was 40.1 ± 27.6 events/hr. Apnea-hypopnea indexes were 38.3 ± 30.6 and 51.9 ± 28.3 events/hr for NREM and REM sleep, respectively. No differences were detected in obstructive respiratory event duration or degree of desaturation between REM and NREM sleep. Additionally, no difference in blood pressure (systolic and diastolic) was detected between REM and NREM sleep during obstructive events and post-obstructive event period. Simple linear regression identified history of hypertension as a predictor of increased systolic blood pressure during obstructive events and post-obstructive event period in both rapid eye movement and non-rapid eye movement sleep. Oxygen desaturation index was also a predictor of increased systolic blood pressure during obstructive events and post-obstructive event period in REM sleep. When obstructive event duration and the degree of desaturation were comparable, no difference in blood pressure was found between REM and NREM sleep during obstructive events and post-obstructive event period.

## Introduction

Obstructive sleep apnea (OSA) is a common sleep disorder that occurs during rapid eye movement (REM) and non-REM (NREM) sleep^1^. REM sleep constitutes approximately 25% of the total sleep time in healthy adults. Interaction of several factors leads to longer duration and greater severity of oxygen desaturation during obstructive events in REM sleep than in NREM sleep. REM-predominant or REM-only OSA occurs in one-third of patients presenting to clinical sleep disorder services^2,3^.

It has been proposed that REM-OSA has more deleterious effects on the cardiometabolic system than does NREM-OSA. During REM sleep, sympathetic activity increases, and cardiovascular instability is prominent^4^. Recent studies suggest that REM-only OSA with an apnea-hypopnea index (AHI) > 15 events/h or NREM AHI < 5 events/h is independently associated with prevalent and incident hypertension, as well as with non-dipping of the nocturnal blood pressure (BP)^5,6^. Usually, autonomic functions are stable during NREM sleep; however, significant instability in autonomic functions can be observed during REM sleep^7^. The baroreflex gain is heightened in response to BP increments during NREM sleep, and the increased baroreflex gain probably helps to maintain stable low BP during NREM sleep^7^. On the other hand, the instability in autonomic functions during REM sleep produces an imbalance between parasympathetic and sympathetic influences, which results in sudden and abrupt changes in BP^7^.

An earlier study found that the immediate post-apnea period was associated with increased mean arterial pressure, which was preceded by a rise of muscle sympathetic nerve activity^8^ however, none of the studied patients had REM sleep. In addition, an association of rapid oscillations in BP with apneic events has been observed during sleep in OSA patients^9–16^. Mean BP decreases during normal sleep but can increase during sleep in OSA patients^14,17^. However, previous studies have reported some conflicting findings regarding the effect of sleep states on the cardiovascular changes during obstructive respiratory events, and not all studies have assessed BP changes during obstructive events in REM sleep^18–20^. Additionally, most previous studies did not account for obstructive respiratory events duration and the degree of desaturation on BP changes in different sleep stages. Therefore, further studies are needed to examine the effects of OSA on BP in REM and NREM sleep.

A non-invasive method that does not cause sleep disturbances is needed to assess BP continuously during different sleep stages to eliminate the effects of arousal and sleep disturbances on BP. Notably, a direct method based on the relationship between BP and the pulse wave velocity (PWV) can be used to determine BP noninvasively. Good correlations have been found between cardiovascular parameters and BP assessed using the pulse transit time (PTT) in patients with OSA^21^, and BP measured using the PTT is comparable with that assessed by reference methods^22–25^.

Because REM sleep is accompanied by a larger sympathetic surge and more accentuated hemodynamic fluctuations than in NREM sleep, the apnea-related rise in BP might be higher during REM sleep than during NREM sleep even if apnea duration oxygen desaturation were comparable. Therefore, we hypothesized that BP during and immediately after obstructive events is higher in REM sleep than in NREM sleep. To this end, this study non-invasively assessed BP during and immediately after REM and NREM obstructive events using the PTT-based method.

## Methods

### Subjects

This study recruited consecutive continuous positive airway pressure-naïve OSA patients (aged 18 years and above) who were diagnosed using overnight polysomnography (PSG) (SOMNOmedics GmbH, Randersacker, Germany) between January and July 2018. Patients with previous surgical procedures for treatment of snoring or OSA, daytime hypercapnia (PaCO2 > 45 mmHg), congestive heart failure, neuromuscular diseases, or home oxygen therapy were excluded from this study. Additionally, all subjects with arrhythmias, which may influence the electrocardiogram (ECG) and plethysmography signals and thereby impede an accurate detection of the PTT, were also excluded from the study. Initially, 44 OSA patients were recruited, but 12 patients were excluded because REM sleep was not achieved, or they did have REM sleep duration of ≥20 min. Therefore, 32 patients with both NREM and REM sleep periods were included in this study.

The study was approved by the ethics committee of the College of Medicine at King Saud University, and written informed consent was obtained from all participants. The used methods were carried out in accordance with the Declaration of Helsinki and the relevant guidelines and regulations.

### Sleep study

All participants underwent pulmonary function tests and arterial blood gases. Additionally, all undertook type I attended polysomnography, and the following physiological parameters were monitored: six leads of EEG (F4 – M1, C4 – M1, O2 – M1, F3 – M2, C3 – M2, and O1 – M2), electrooculography (EOG), chin electromyography (EMG), electrocardiography (ECG), oxygen saturation, chest and abdominal wall movements, air flow (thermistor and nasal pressure), and sleep position assessed using Somnoscreem plus^TM^ diagnostic equipment (SOMNOscreen plus^TM^, Randersacker, Germany).

Raw data were scored manually according to established scoring criteria^26^. Apnea was scored when a drop in the peak thermal sensor excursion of ≥90% of the baseline lasted for ≥10 seconds. Hypopnea was considered when the nasal pressure signal decreased by ≥30% for at least ≥10 seconds, resulting in a ≥ 3% decrease in oxygen saturation from the pre-event baseline or arousal. The AHI, which was computed by dividing the number of episodes of apneas and hypopneas by total sleep time in hours, is expressed as events/hour. The duration of the obstructive events was measured from the first breath that is clearly reduced to the beginning of the first breath that approximates the baseline amplitude^26^. Obstructive AHI was calculated during REM and NREM sleep. The desaturation index, which was assessed by dividing the number of desaturations (drop in oxygen of ≥3%) by the total sleep time, is expressed as events per hour of sleep.

Arousal was scored during sleep stages N1, N2, N3, or REM when there was an abrupt shift of EEG frequency (including alpha, theta, and/or frequencies greater than 16 Hz [but not spindles]), which lasted at least 3 seconds, with at least 10 seconds of stable sleep preceding the change^26^. REM arousal scoring requires a concurrent increase in submental EMG lasting at least 1 second^26^.

### Blood pressure measurement

BP was measured indirectly using a validated method based on the PTT. PWV and BP were assessed using the DOMINO-Software (DOMINO 2.2.0 supplied with the SOMNOscreen plus^TM^, Randersacker, Germany). ECG and the finger photoplethysmography curve were recorded with the SOMNOscreen^TM^ polysomnography device^22,25,27^. The SOMNOscreen plusTM noninvasive blood pressure measurement has been validated according to the European Society of Hypertension (ESH) protocol^27^.

SOMNOscreen plus^TM^, which allows continuous (beat-to-beat) and noninvasive BP monitoring, includes a finger photoplethysmograph that additionally provides oxygen saturation measurement. Two bipolar ECG electrodes were attached parasternally at the second right and fifth left intercostal spaces. A ground electrode was fixed to the lower limb. The finger plethysmography curve was recorded with the SOMNOscreen^TM^ polysomnography device^22,25^.

BP was calculated using the DOMINO software with the one-point calibration based on a PWV-blood-pressure relation^22,27^. It has been demonstrated that the value obtained by this calibration remains constant^22^. Continuous BP estimation was based on the beat-to-beat determination of PTT, calculated as the interval between the ECG R-waves and the detection of the corresponding pulse wave (revealed from the finger photoplethysmography signal) at the peripheral site.

SOMNOmedics uses the innovative PTT method for the determination of the blood pressure by means of a patented algorithm^28^. The calculation of the systolic and diastolic blood pressure is based on a non-linear correlation between blood pressure (in mmHg) and PTT (in ms)^22^. Systolic BP and diastolic BP readings were calculated based on the relationship between BP levels and PTT^27^.

BP was assessed during pre-obstructive events (10 sec of regular breathing pattern immediately preceding the obstructive event) (this will be called “Quiet Sleep” BP), obstructive events, and post-obstructive events (average of the 3 consecutive peak measurements within 15 sec of event termination)^29^. Arterial pressure was measured on a beat-by-beat basis throughout the obstructive events and in the immediate post-obstructive event period during NREM and REM. The average values of BP during the pre-obstructive events, during obstructive events (both apneas and hypopneas), and in the immediate post-obstructive event period were used in the analysis. Additionally, BP was measured for 10 min while awake in the supine position in bed using the same method, and the average awake BP was also used in the analysis (this will be called “Awake” BP).

### Statistical analysis

Data are expressed as means ± SD for continuous data and n (%) for categorical data. The student t-test was used to compare continuous data if the normality assumption was met; otherwise, the Mann-Whitney U test was used. Analysis of variance was used for normally distributed data for comparison of three or more continuous variables; if the normality test failed, Kruskal–Wallis test by ranks was used. The χ2 test was used to compare categorical variables.

Simple linear regression was used to determine associations between changes in systolic BP during obstructive and post-obstructive events and independent factors. Independent variables included age, body mass index (BMI), desaturation index, arousal index, obstructive event duration, AHI, and history of hypertension.

Based on a previous study that assessed systolic BP changes after apnea^30^, and demonstrated a change in systolic BP of 8 mmHg (SD 19.5) in REM sleep and a change of 6 mmHg (SD 15.5) in NREM sleep, we estimated the needed sample size to detect similar differences as n = 27 based on the following equation (n = (Z-score)² × StdDev²/(margin of error)²)^31^.

A *P* of ≤0.05 was considered significant. Statistical analyses were performed using SPSS ver. 21.0 (Chicago, IL, USA).

### Research involving human subjects

The study protocol was approved by the institutional review board at King Saud University, and informed consent was obtained from all the participants prior to inclusion in this study. The used methods were carried out in accordance with the Declaration of Helsinki and the relevant guidelines and regulations.

### Informed consent

Written informed consent was obtained from all participants.

## Results

### Clinical and demographic characteristics of patients

The study included 32 patients (males: 50%) with a mean age of 50.2 ± 12.1 years and a BMI of 34.1 ± 7.9 kg/m^2^. Table 1 shows the demographic and general information of the studied patients. Among the recruited patients, 10 (31.3%) had hypertension. Table 2 shows the PSG findings of patients. The mean AHI was 40.1 ± 27.6 events/hr. AHI-NREM was 38.3 ± 30.6 events/hr, and AHI-REM was 51.9 ± 28.3 events/hr. BP was higher during wakefulness than during sleep; however, the difference did not reach statistical significance.

**Table 1:** Demographic and general information of patients.

Variables	Mean ± SD/n (%)
Age (years)	50.2 ± 12.1
Body mass index (kg/m^2^)	34.1 ± 7.9
Sex (male)	16 (50)
Epworth Sleepiness Scale	11.9 ± 7
Systolic BP (awake supine in bed)	129 ± 19.1
Diastolic BP (awake supine in bed)	76.6 ± 11
**Arterial blood gases**	
pH	7.4 ± 0.1
PaCO_2_ (mmHg)	39.5 ± 5.4
PaO_2_ (mmHg)	76.8 ± 21.9
HCO_3_ (mmol/L)	25.5 ± 4.1
FEV_1_/FVC (%)	88.8 ± 7.8
FVC (% predicted)	80.6 ± 28
FEV_1_ (% predicted)	86.5 ± 30.2
**Comorbidities**	
Hypertension	10 (31.3)
Ischemic heart disease	0 (0)
Diabetes mellitus	9 (29)
Compensated heart failure	0 (0)
Stroke	0 (0)
Bronchial asthma	3 (9.4)
Hypothyroidism	5 (15.6)
Hypercholesterolemia	10 (31.3)
**OSA Severity**	
Mild OSA	4 (12.5)
Moderate OSA	12 (37.5)
Severe OSA	16 (50)

A total of 32 patients participated in this study. BP, blood pressure; OSA, obstructive sleep apnea; FVC, forced vital capacity; FEV, forced expiratory volume.

**Table 2:** Polysomnographic findings of patients.

Variables	Mean ± SD/n (%)
Time in bed	381.2 ± 79.7
Total sleep time	290.5 ± 65.4
Sleep efficiency (%)	76 ± 13.7
Stage N1 (%)	8.3 ± 7.3
Stage N2 (%)	63.4 ± 12.9
Stage N3 (%)	13.7 ± 13.2
Stage REM (%)	13.4 ± 5.5
Apnea hypopnea index (AHI) (events/hr)	40.1 ± 27.6
Obstructive apnea index (events/hr)	3 ± 6.1
Obstructive hypopnea index (events/hr)	35.9 ± 23.6
Desaturation index (desaturations/hr)	23 ± 22.8
Time with SpO_2_ < 90% (mins)	12.8 ± 17.8
Lowest recorded SpO_2_ (%)	82 ± 10.5
Mean nocturnal SpO_2_ (%)	94.1 ± 2.8
Arousal index (arousals/hr)	33.2 ± 20.1

A total of 32 patients participated in this study. REM, rapid eye movement; NREM, non-REM.

### Blood pressure during the obstructive and post-obstructive events

**Figure 1:**
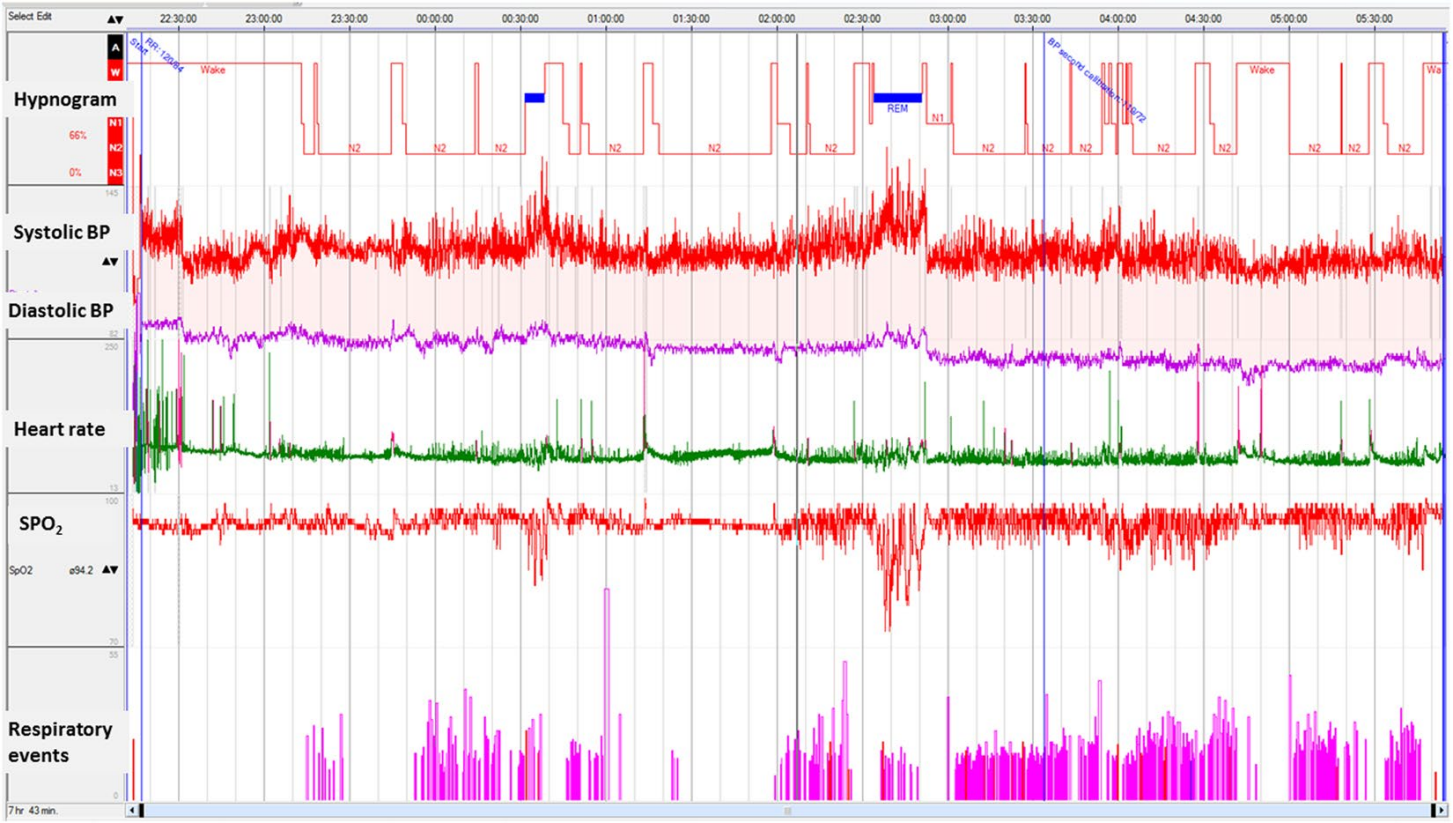
A histogram of a patient showing an increase in blood pressure during REM sleep with significant desaturation.

**Figure 2:**
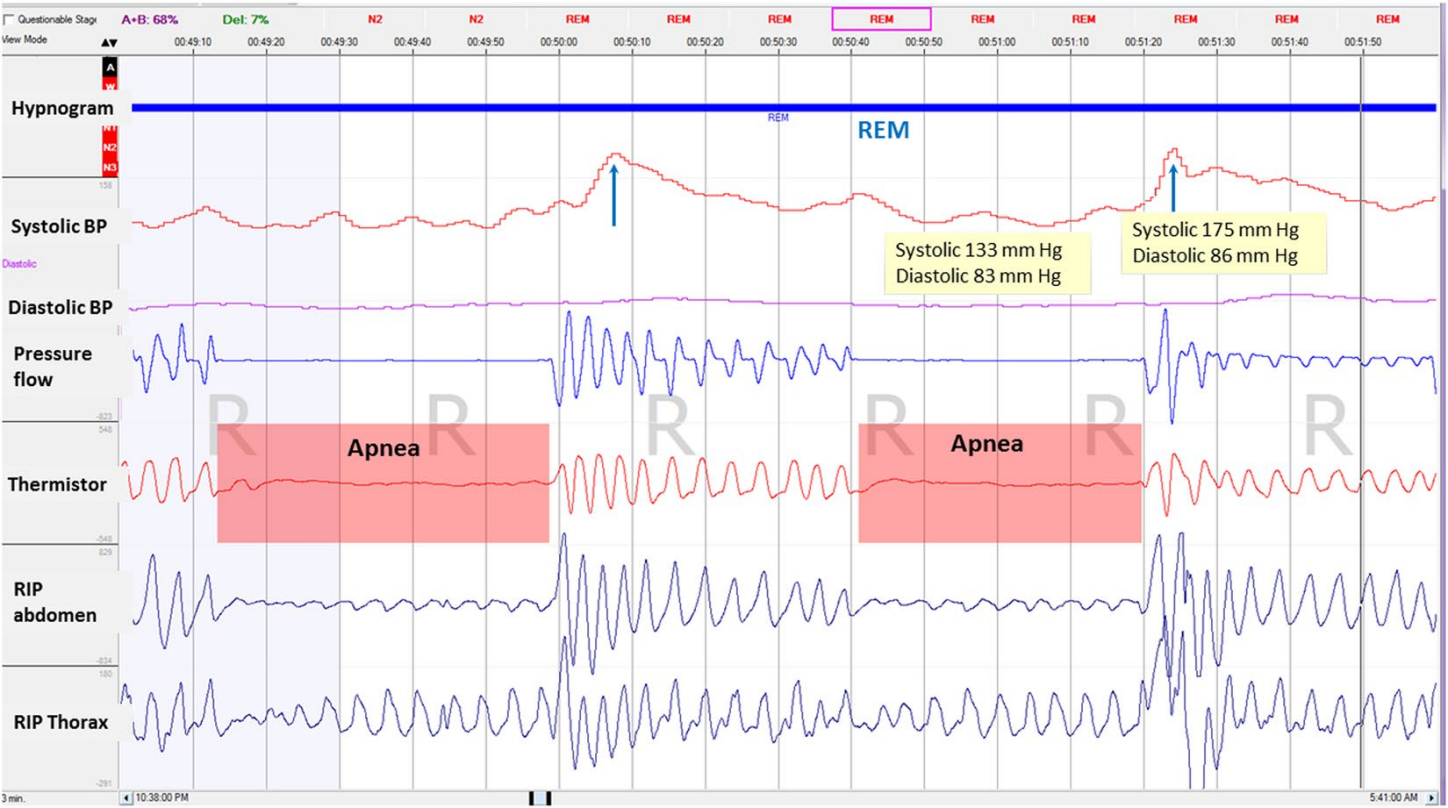
A zoomed epoch of 6 minutes during REM sleep showing an increase in blood pressure in the post-obstructive event period (blue arrows). REM: rapid eye movement.

Obstructive events in NREM and REM were available for analysis in all patients (n = 32). Figure 1 demonstrates a histogram of a patient showing an increase in BP during REM sleep with significant desaturation and Fig. 2 displays a zoomed epoch of 6 minutes during REM sleep showing an increase in blood pressure in the post-obstructive event period. REM, rapid eye movement.

Table 3 shows the mean systolic and diastolic BP assessed using the PTT method during wakefulness, sleep, REM and NREM obstructive respiratory events, and NREM and REM post-obstructive events. No statistically significant difference in BP (both systolic and diastolic) was detected between REM and NREM during obstructive events (130.3 ± 17/81.1 ± 11.1 mmHg versus 126.8 ± 16.3/76.9 ± 10.3 mmHg) or post-obstructive events (130 ± 17.4/80.8 ± 11.4 mmHg versus 127 ± 15.5/78.3 ± 9). Additionally, no differences were detected in obstructive respiratory event duration or time spent with SPO_2_ < 90% between REM and NREM sleep (23.8 ± 11.2 sec versus 20.8 ± 4.9 sec and 13.8 ± 19.4 min versus 11.9 ± 22.0 min, respectively).

**Table 3:** Comparison of blood pressure during obstructive and post-obstructive respiratory events between REM and NREM sleep.

Variable	Awake	Quiet Sleep	During the obstructive event			Post-obstructive event		
			REM	NREM	p-value	REM	NREM	p-value
Systolic BP	129 ± 19.1	122.7 ± 15.7	130.3 ± 17	126.8 ± 16.3	0.4	130 ± 17.4	127 ± 15.5	0.3
Diastolic BP	76.6 ± 11	76.8 ± 10.4	81.1 ± 11.1	76.9 ± 10.3	0.15	80.8 ± 11.4	78.3 ± 9	0.3
Mean apnea -hypopnea duration (Sec)			23.8 ± 11.2	20.8 ± 4.9	0.6			
Time with SpO2 < 90% (Min)			13.8 ± 19.4	11.9 ± 22.0	0.7			

Systolic BP increased by approximately 8 mmHg and 4 mmHg during obstructive events in REM and NREM events, respectively, compared with that in quiet sleep. Systolic BP increased by approximately 7 mmHg and 4 mmHg during the post-obstructive event period in REM and NREM sleep, respectively, compared with that in quiet sleep.

In a sub-group analysis, we analyzed BP data for patients without hypertension (n = 22) (Table 4). As for the whole group, no statistically significant difference in BP (both systolic and diastolic) was detected between REM and NREM during obstructive events (124.7 ± 12.6/79.2 ± 9.7 mmHg versus 121.3 ± 13.3/74.6 ± 9.3 mmHg) or post-obstructive events (121.2 ± 13.2/79.7 ± 9.8 mmHg versus 117.6 ± 13.3/74.7 ± 9.5). Similarly, no differences were detected in obstructive respiratory event duration or time spent with SPO_2_ < 90% between REM and NREM sleep. Systolic BP increased by approximately 7 mmHg and 4 mmHg during obstructive events in REM and NREM events, respectively, compared with that in quiet sleep. Systolic BP increased by approximately 4 mmHg during the post-obstructive event period in REM and no changes in systolic BP during NREM sleep, compared with that in quiet sleep.

**Table 4:** Comparison of blood pressure during obstructive and post-obstructive respiratory events between REM and NREM sleep in patients without hypertension (n = 22).

Variable	Awake	Quiet Sleep	During the obstructive event (n = 22)			Post-obstructive event		
			REM	NREM	p-value	REM	NREM	p-value
Systolic BP	123.4 ± 17.2	117.6 ± 13.3	124.7 ± 12.6	121.3 ± 13.3	0.8	121.2 ± 13.2	117.6 ± 13.3	0.4
Diastolic BP	74.4 ± 10.2	74.7 ± 9.5	79.2 ± 9.7	74.6 ± 9.3	0.2	79.7 ± 9.8	74.7 ± 9.5	0.1
Mean apnea -hypopnea duration (Sec)			23.1 ± 12.1	19.9 ± 6.1	0.3			
Time with SpO2 < 90% (Min)			12.9 ± 19.4	11.3 ± 18.5	0.7			

Subsequently, we categorized patients into those with severe (AHI ≥ 30 events/hr) and mild to moderate OSA (AHI < 30 events/hr) (Table 5). During NREM sleep obstructive events, patients with severe OSA (AHI > 30) had higher systolic pressure than did those with mild to moderate OSA (130.1 ± 17.3 mmHg vs. 123.1 ± 14.7 mmHg, p = 0.05). However, during REM sleep obstructive events, no difference in BP was detected between patients with mild to moderate OSA and those with severe OSA (130.2 ± 18.6 mmHg vs. 130.7 ± 12.3 mmHg). In the post-obstructive event period, no difference in BP was detected in both REM and NREM events between patients with severe and mild to moderate OSA.

**Table 5:** Comparisons between patients with severe and mild to moderate OSA in REM and NREM sleep.

Variables	Awake	Sleep	REM-OSA ≥ 30 (n = 24)	REM-OSA < 30 (n = 8)	p-value	NREM-OSA ≥ 30 (n = 16)	NREM-OSA < 30 (n = 16)	p-value
Mean obstructive event duration	129 ± 19.1	122.7 ± 15.7	22.9 ± 8.2	26.2 ± 18.1	0.7	20.8 ± 5.7	20.9 ± 4.1	0.9
Time with SpO2 < 90% (min)	76.6 ± 11	76.8 ± 10.4	13.8 ± 19.4	7.3 ± 4.5	0.8	15.5 ± 23.8	2.7 ± 4.1	0.06
**During the obstructive event**								
Systolic BP during obstructive event			130.2 ± 18.6	130.7 ± 12.3	0.96	130.1 ± 17.3	123.1 ± 14.7	0.05
Diastolic BP during obstructive event			82.6 ± 10.2	76.8 ± 13	0.2	79.9 ± 11.7	73.6 ± 7.6	0.3
**Post-obstructive event**								
Systolic BP Post-Events			129.4 ± 19.2	131.5 ± 13.5	0.8	131.8 ± 17.8	124.6 ± 17.4	0.4
Diastolic BP Post-Events			81.9 ± 10.6	77.8 ± 13.9	0.4	82.8 ± 10.7	75.4 ± 12.4	0.1

### Determinants of changes in blood pressure during REM and NREM obstructive events

Simple linear regression analysis for the prediction of systolic BP during obstructive events in REM and NREM is shown in Table 6. During obstructive events in REM sleep, oxygen desaturation index, awake BP, and hypertension were the significant predictors of large changes in systolic BP. However, during obstructive events in NREM, awake BP and hypertension were the only significant predictors of large changes in BP.

**Table 6:** Linear regression analysis to predict systolic blood pressure during REM and NREM sleep (n = 32). (Enter Method).

During REM sleep obstructive events				
Variables in the Equation	β Coefficient	Standard Error	95% C.I.	P-Value
Age (years)	0.369	0.276	−0.199–0.937	0.193
AHI-REM (events/hr)	−0.007	0.117	−0.249–0.234	0.950
Desaturation Index (desaturations/hr)	0.422	0.166	0.08–0.764	0.018
Arousal Index (arousals/hr)	0.243	0.158	−0.082–0.569	0.136
Mean obstructive event duration (Seconds)	0.427	0.705	−1.035–1.888	0.551
Known hypertension	16.839	6.210	4.049–29.629	0.012
Awake blood pressure	0.644	0.120	0.397–0.89	<0.001
Sex (Male)	1.052	5.745	−10.698–12.802	0.856
Diabetes mellitus	4.854	6.172	−7.809–17.517	0.438
**During NREM sleep obstructive events**				
Age (years)	0.403	0.242	−0.092–0.899	0.106
AHI-NREM (events/hr)	0.176	0.114	−0.059–0.411	0.136
Desaturation Index (events/hr)	0.254	0.166	−0.087–0.596	0.138
Arousal Index (arousals/hr)	0.062	0.147	−0.239–0.364	0.674
Mean obstructive event duration (Seconds)	−0.673	0.618	−1.94–0.593	0.285
Known hypertension	18.438	5.600	6.968–29.908	0.003
Awake blood pressure	0.710	0.070	0.567–0.853	<0.001
Sex (Male)	2.127	5.517	−9.141–13.394	0.703
Diabetes mellitus	6.246	6.155	−6.362–18.854	0.319

Simple linear regression analysis for the prediction of systolic BP during the post-obstructive event period in REM and NREM is shown in Table 7. During both REM and NREM sleep, history of hypertension and awake BP were the predictors of large changes in BP, and during REM sleep, oxygen desaturation index was a predictor of large changes in BP.

**Table 7:** Linear regression analysis to predict systolic blood pressure during post-events arousal REM (n = 32). (Enter Method).

During post obstructive events arousal in REM sleep				
Variables in the Equation	β Coefficient	Standard Error	95% C.I.	P-Value
Age (years)	0.345	0.289	−0.25–0.94	0.244
AHI-REM (events/hr)	−0.029	0.122	−0.28–0.222	0.813
Desaturation Index (events/hr)	0.440	0.173	0.084–0.796	0.017
Arousal Index (arousals/hr)	0.214	0.167	−0.13–0.557	0.212
Mean obstructive event duration during REM (Seconds)	0.246	0.724	−1.255–1.747	0.737
Known hypertension	17.761	6.441	4.495–31.027	0.011
Awake blood pressure	0.699	0.104	0.486–0.913	<0.001
Sex (Male)	11.298	6.350	−1.754–24.351	0.087
Diabetes mellitus	−1.710	7.841	−17.894–14.474	0.829
**During post obstructive events arousal in NREM sleep**				
Age (years)	0.467	0.238	−0.02–0.954	0.059
AHI-NREM (events/hr)	0.116	0.117	−0.124–0.355	0.330
Desaturation Index (events/hr)	0.161	0.170	−0.187–0.51	0.350
Arousal Index (arousals/hr)	−0.011	0.148	−0.313–0.292	0.943
Mean obstructive event duration (Seconds)-NREM	−0.620	0.620	−1.891–0.651	0.326
Known hypertension	16.544	5.810	4.644–28.445	0.008
Awake blood pressure	0.705	0.074	0.552–0.857	<0.001
Sex (Male)	5.643	5.577	−5.763–17.049	0.320
Diabetes mellitus	2.377	6.570	−11.104–15.859	0.720

## Discussion

This study assessed acute changes in BP during obstructive and post-obstructive respiratory events in adults. The findings revealed increased BP during obstructive events and post-obstructive event period in both REM and NREM sleep; however, the differences in BP between REM and NREM were not statistically significant during the obstructive events or the post-obstructive event period. Nevertheless, the obstructive event duration and degree of desaturation were similar in both REM and NREM sleep. Although the difference in BP did not reach statistical significance, systolic BP was higher by approximately 3.5 mmHg in REM sleep compared to NREM sleep (Table 3), and it was sustained during post-obstructive event; the magnitude of the difference is clinically important though the lack of significance might be due to the relatively low sample size. Simple linear regression identified history of hypertension as a predictor of increased BP during obstructive events and the post-obstructive event period in both REM and NREM sleep.

Changes in BP during sleep in OSA patients are rapid with large swings; therefore, suitable methods are needed to monitor fast changes in BP, such as beat-by-beat measurement of BP. The PTT and PWV method is among the most common applications of beat-to-beat BP recordings^22,24,25,32^. The beat-by-beat measurement of BP allows accurate detection of the very short-term variability in BP, although it may be less accurate in the assessment of absolute BP readings^33^.

Previous studies have demonstrated increased arterial BP following obstructive apnea in adult OSA patients^18–20^. Nevertheless, some conflicts exist in the reported data regarding the effect of sleep states on the cardiovascular changes during obstructive respiratory events, and not all studies have assessed BP changes during obstructive respiratory events in REM sleep. Jelic *et al*. found no differences in BP between sleep states; however, mean systolic BP was significantly lower during NREM apnea than during awake state but was significantly higher after apnea than during awake or apnea^30^. They also found that systolic BP changes during REM and NREM sleep had similar patterns with a reduction from the awake state to occurrence of apnea and an increase in the post-apnea period. In addition, Garpestad *et al*. demonstrated a higher BP elevation during post-obstructive events in both NREM and REM sleep, and the elevation was higher during REM sleep than during NREM sleep^18^. Similarly, Okabe *et al*. showed a greater increase in BP in the post-obstructive event period during REM in adult OSA patients, and the obstructive events in REM sleep were longer and associated with greater oxygen desaturation than those in NREM sleep^34^. A later study in children with OSA (included only respiratory events ≥10 s in duration) also revealed that obstructive events were longer and associated with greater desaturation in REM sleep; however, the overall cardiovascular change was larger in NREM^29^. In the present study, BP was higher during obstructive events and post-obstructive event period in both REM and NREM sleep than in quiet sleep, although this difference did not reach statistical significance.

Several mechanisms have been proposed to explain the increase in BP during or after an obstructive respiratory event. The obstructive event during sleep is associated with increased negative intrathoracic pressure, hypoxia, hypercapnia, and arousal responses, and these changes induce drastic increases in sympathetic activity^35^. During an obstructive event, the inhibitory effect of the thoracic afferents is absent, thus potentiating sympathetic activation^36^. As a result, vasoconstriction develops and causes a significant elevation in BP. At the end of the obstructive event, breathing resumes and apnea-induced restrictions on stroke volume and heart rate are abruptly eliminated, allowing ejection of the augmented cardiac output into a peripheral vascular bed that has been restricted by the sympathetic induce vasoconstriction; thus, a transient increase in BP might be induced in the immediate post-apnea period accordingly^7,36^.

Sleep stage may also affect BP in patients with OSA because the duration and severity of obstructive events vary with sleep stages. Obstructive events are less frequent during slow-wave sleep, and obstructive respiratory events are usually longer and associated with worse hypoxemia during REM sleep^37,38^. However, no significant difference was detected in BP during obstructive events between REM and NREM sleep in this study (Table 3). This could be related to the fact that no differences in the duration of obstructive events or time spent with SPO_2_ < 90% between REM and NREM sleep were detected in the present study. A previous study assessed the relationship between obstructive event duration and concomitant BP changes in nine patients with OSA^39^. As the duration of the obstructive event increased from 10 sec to greater than 30 sec, BP increased^39^. Another study demonstrated that OSA patients with a longer obstructive event duration had more severe hypertension when compared with patients with a shorter event duration^40^. Garpestad *et al*. reported that during REM sleep, prolongation of apnea duration resulted in lower oxygen desaturation and was associated with further increases in post-apneic BP^18^. Another interesting finding of this study is BP response during obstructive events in REM sleep. BP was similar in mild-to moderate and severe OSA in REM sleep, while BP response was lower in mild-to-moderate OSA in NREM sleep. This finding may mean that BP increase starts already with lower degree of OSA during REM sleep while the changes are notable first in severe OSA in NREM sleep. Further studies are needed to confirm this finding.

A plausible explanation for the reported relationship between BP and obstructive event duration is that, the longer the duration of the event, the higher the hypercapnia and the lower the hypoxemia^39^. Hypercapnia and hypoxemia synergistically act to increase sympathetic nerve activity^41^. The current study findings that oxygen desaturation was a predictor of increased BP during REM sleep obstructive events concur with the above explanation. Cyclical changes in BP have been observed during repeated intermittent hypoxemia in awake patients with OSA^34^. Moreover, increased ventilatory response to hypoxia may lead to a larger inspiratory effort upon resumption of breathing, which could result in increased BP^29,34^.

This study has some strengths. First, the study included patients with both REM and NREM obstructive events, thus allowing the comparison between REM and NREM sleep in the whole study group. Second, the study used a well-validated noninvasive and continuous beat-to-beat measurement method. However, this study does have several limitations. First, although we calculated the sample size, the studied sample remains relatively small. Therefore, studies with a larger sample size are needed to allow better detection of differences in BP. Second, the current findings do not agree with our hypothesis and cannot explain why REM-OSA is associated with high hypertension. This could be related to the fact that mean obstructive respiratory events duration and the degree of oxygen desaturation were not different in both REM and NREM respiratory events. Additionally, the current findings challenge recent data showing that REM-OSA is the main culprit for hypertension in patients with OSA, which calls for more research to verify the association between REM-OSA and hypertension. Third, the current paper has no control group. Therefore, future studies should include hypertensive patients with and without OSA.

In summary, this study revealed increased BP during obstructive events and in the post-obstructive event period in both REM and NREM sleep. However, no statistically significant differences in BP between REM and NREM sleep were detected. The lack of difference in BP between obstructive events in REM and NREM sleep could be related to the fact that obstructive event duration and the degree of desaturation were comparable in REM and NREM obstructive events in the study group. Future studies should assess the relationship between apnea duration in REM and NREM sleep and changes of BP as well as prevalent and incident hypertension.

Simple linear regression identified history of hypertension as a predictor of increased BP during obstructive events and in the post-obstructive event period in both REM and NREM sleep. Further studies with non-intrusive technologies are needed to enable frequent and accurate assessments of vascular function during different stages.
